# Impact of somatic copy number alterations on the glioblastoma miRNome: miR‐4484 is a genomically deleted tumour suppressor

**DOI:** 10.1002/1878-0261.12060

**Published:** 2017-05-24

**Authors:** Zahid Nawaz, Vikas Patil, Sivaarumugam Thinagararjan, Soumya A. Rao, Alangar S. Hegde, Arimappamagan Arivazhagan, Vani Santosh, Kumaravel Somasundaram

**Affiliations:** ^1^ Department of Microbiology and Cell Biology Indian Institute of Science Bangalore Karnataka India; ^2^ Department of Neurosurgery Sri Satya Sai Institute of Higher Medical Sciences Bangalore Karnataka India; ^3^ Department of Neurosurgery National Institute of Mental Health and Neuro Sciences Bangalore Karnataka India; ^4^ Department of Neuropathology National Institute of Mental Health and Neuro Sciences Bangalore Karnataka India

**Keywords:** glioblastoma, growth suppression, miR‐4484, miRNome, somatic copy number alterations

## Abstract

Glioblastoma (GBM) is the most frequent and most malignant primary brain tumour in adults. GBMs have a unique landscape of somatic copy number alterations (SCNAs), with the concomitant appearance of numerous driver amplifications and deletions. Here, we examined the genomic regions harbouring SCNAs and their impact on the GBM miRNome. We found that 40% of SCNA events covering 70–88% of the genomically altered regions, as identified by GISTIC and RAE algorithms, carried miRNA genes. Of 1426 annotated mature miRNAs analysed, ~ 14% (*n* = 198) were mapped to such fragile loci. Further, we identified an intragenic miRNA, miR‐4484 located on chromosome‐10, as a deleted and downregulated miRNA in GBM. miR‐4484 exhibited a strong positive correlation with the expression of its host gene *uroporphyrinogen III synthase* (*UROS*), thereby indicating that the loss of miR‐4484 is a codeletion event in GBM. Overexpression of miR‐4484 reduced the colony‐forming ability and suppressed the migratory capacity of glioma cells. Analysis of the RNA‐seq‐derived transcriptome upon exogenous miR‐4484 overexpression in conjunction with an integrative bioinformatics approach revealed several putative targets of miR‐4484. Unbiased functional enrichment of these targets through DAVID identified a cohort of important gene ontology terms, which possibly explain the functional role of miR‐4484 in gliomagenesis. Selected targets were validated and, importantly, were found to be upregulated in GBM. In brief, our study identified a panel of miRNAs that are likely to be regulated by genomic deletions and amplifications. Further, miR‐4484 was found to be deleted and acts as a tumour suppressor miRNA in GBM.

**Abbreviations**
GBM: glioblastoma
GISTIC: Genomic Identification of Significant Targets in Cancer
KEGG: Kyoto Encyclopedia of Genes and Genomes
RAE: Relative Absolute Error
TCGA: The Cancer genome Atlas
UROS: uroporphyrinogen III synthase

## 1 Introduction

The most malignant variant of the intracranial tumours is constituted by glioblastomas (GBMs), which in fact represent the most prevalent primary brain tumours in adults. Owing to their largely infiltrative growing pattern, they easily preclude definitive surgical resection and portend a dismal clinical outcome on account of their invariably aggressive nature. The therapeutic modalities and interventions that are being employed currently comprise of tumour resection followed by radiotherapy and temozolomide treatment. These approaches for GBM management fail to bring the much needed respite to the patients, as the survival of GBM continues to lurk around 12–15 months (Stupp *et al*., 2005; Wen and Kesari, 2008). Thus, the current scenario of GBM management is more palliative rather than being curative. Such a poor prognosis in GBM is credited to the therapeutic resistance and tumour recurrence, which are common place events in GBM and occur after the surgical removal of the tumour. In essence, the recurrence of GBM relies on the caveat in our therapeutic approach as it is technically impossible to resect every bit of the tumour precisely, thereby leaving inevitably some malignant cells at the site of the primary tumour. Hypothetically, these cells are enriched in cancer stem cells, which evade chemotherapy and advance to propel the recurrent tumours.

As a well‐known fact, the initiation and progression of cancer is closely associated with the accumulation of numerous genetic alterations in the genome. The interplay between these alterations allows cancer to evolve clonally into an independently overwhelming entity, through the deactivation of tumour suppressor genes (loss‐of‐function mutations) and activation of oncogenes (gain‐of‐function mutations). One of the important genetic lesions that plays a pivotal role in tumorigenesis is the somatic copy number alterations (SCNAs). SCNAs encompass sequences that have different copy numbers in an individual's germline DNA and in the DNA of a clonal subpopulation of cells, which happen to be malignant (Beroukhim *et al*., 2010). In other words, the SCNAs involve genomic gains (amplifications, duplications) or losses (deletions) that alter the diploid status of a particular locus, thereby disrupting the balanced genome. The cancer genome is incessantly shaped by such modifications in its structure, and this process enables it to adapt, evolve and become more distorted. Moreover, these SCNAs also add tremendous variability to the genome, making the treacherous evolution of cancer further intricate, as some of the copy number aberrations could be just the consequence of the earlier occurred events and may not contribute to tumorigenesis. SCNAs impact the cancer phenotype through the amplification of oncogenes and deletions of tumour suppressor genes. SCNAs form the sources of genomic instability and structural dynamism, which are the important classifiers of cancer cells. SCNAs in particular are the most intriguing form of genetic variations to study in cancer, as they alter the dosage of a gene product independent of the transcription factors and other controls. Analyses of cancer genome by cytogenetic studies and more recently by array hybridization profiling‐based methodologies have assisted in the identification of recurrent alterations unique to various cancers as well as some common alterations (Beroukhim *et al*., 2010; Zack *et al*., 2013). In understanding the role of CNAs, it becomes imperative to characterize the CNAs as the driver events in a particular malignancy and distinguish them from the passenger CNAs that would have been imbued into the genome during cancer evolution and possibly do not contribute towards it (Zack *et al*., 2013).

In a close resemblance to other malignancies, GBMs are also afflicted by a multitude of genetic and epigenetic lesions which results in the heterogeneity observed in GBM. The study carried out by The Cancer Genome Atlas (TCGA) in 2008 described the different events, such as multiple chromosomal aberrations, nucleotide substitutions and epigenetic modifications that drive the malignant transformation in GBM (Bartel, 2004, 2008). In fact, GBMs reveal a largely deranged karyotype upon cytogenetic analysis with multiple numerical and structural chromosomal aberrations imbued in it. Like other malignancies, GBM is also found to be severely affected by the SCNAs, as there are a myriad of important genes that get derailed owing to copy number aberrations leaving a significant portion of the genome heavily distorted (Cancer Genome Atlas Research 2008). A study carried out by Beroukhim *et al*. (2007), classified these chromosomal aberrations in GBM into two categories: focal and broad, with the broad ones spanning almost the size of either chromosomal arm. Although a lot has been uncovered and revealed about the protein‐coding genes that get affected by copy number alterations in GBM, there is not much information available about the noncoding genes comprising miRNAs, LncRNAs and others. In fact, these noncoding genes can also be influenced by the changes in copy number status of the genome.

As the role of SCNAs regulating the expression of miRNAs in GBM has not been divulged so far, this necessitated us to unveil the GBM‐associated miRNome that gets affected by the somatic copy number alterations. MicroRNAs belong to a class of noncoding RNAs (ncRNAs), which are about 19–25 nucleotides in length and are generated from an endogenous transcript that contains a local hairpin structure (Inui *et al*., 2010; Lee *et al*., 2004). miRNAs constitute one of the largest gene families accounting for ~ 1% of the genome (Kim, 2005). They play a prominent role in fine tuning the expression of protein‐coding genes as they are involved in post‐transcriptional gene silencing. miRNAs function as guide molecules, and base pair with target mRNAs with near‐perfect complementarity, thereby leading to their subsequent cleavage or translational repression. Recent studies have deciphered the role of miRNAs in diverse regulatory pathways that control development, differentiation, apoptosis, cell proliferation and organogenesis (Bartel, 2004; Bushati and Cohen, 2007). In this study, we examined the genomic regions harbouring SCNAs, and by employing an integrated bioinformatics approach, we identified a cohort of miRNAs whose locale corresponds to these fragile sites in the genome, which frequently undergo gene deletions and amplifications. Our study also identified miR‐4484 as a tumour suppressor miRNA which is lost in GBM due to the deletion of its genomic locus. DNA copy number quantitative PCR was used for the validation of focal deletion of the miR‐4484. *In silico* target prediction of miR‐4484 in association with transcriptome analysis by RNA sequencing upon miR‐4484 overexpression lead to the elucidation of its potential targets. miR‐4484 essentially exerts growth‐suppressive function through its inhibitory effect on this cohort of gene targets responsible for producing a malignant phenotype, thereby underscoring the importance of its deletion in GBM development and progression.

## 2 Materials and methods

### 2.1 Patient specimens and biosafety clearance

The GBM tissue specimens were procured from the patients that had undergone surgical resection of GBM (GBM – WHO Grade IV) either at Sri Sathya Sai Institute of Higher Medical Sciences (SSSIHMS) or at National Institute of Mental Health and Neurosciences (NIMHANS), Bangalore, India. The specimens were taken with an informed, written consent from the patients, prior to the initiation of the study, obeying the guidelines laid by the Institutional Ethics Committee (IEC). For comparison sake, we used nontumour brain tissue that was precisely obtained through the anterior temporal lobectomy of intractable epilepsy cases. Both tumour and nontumour control brain samples were snap‐frozen in liquid nitrogen and eventually stored at −80 °C for the purpose of DNA/RNA isolation. A total of 72 GBM samples and 16 control brain samples were used in this study. This study was closely scrutinized and approved by the ethics committee of NIMHANS (NIMHANS/IEC/No. RPA/060/05 dated 29.10.2005) and SSSIHMS (SSSIHMS/IEC/No RPA/001/2005 dated 20.10.2005).

Different methods and experimental procedures adopted in this study are in accordance with the guidelines approved by the Institutional Biosafety Clearance Committee of Indian Institute of Science, Bangalore.

### 2.2 Cell culture

Different glioma cell lines SVG, U87, U138, U251, U343, U373, LN229, LN18 and T98G used in the study were mostly obtained from European Collection of Authenticated Cell Cultures. The cells were grown in Dulbecco's modified Eagle's medium (Sigma, St. Louis, MO, USA) and supplemented with 10% fetal bovine serum (Gibco, ThermoFisher, Bartlesville, OK, USA) along with requisite amounts of penicillin and streptomycin. The cell lines were cultured in a humidified incubator at 37 °C and 5% CO_2_. The medium was changed every two to three days, and the cells were trypsinized at 80–90% confluency.

### 2.3 Genomic DNA isolation and copy number qPCR

Genomic DNA was isolated from cell lines, tumour tissues and normal controls using QIAamp DNA minikit (Qiagen, Germantown, MD, USA) as per the manufacturer's instructions. DNA quality was assessed on a low percentage agarose gel and was quantified by spectrophotometry at 260/280 nm. Copy number analysis of *UROS* and *MIR4484* genes was performed by SYBR green‐based quantitative PCR using DNA‐specific primers of the respective genes (corresponding to the intronic regions of the genes), such that they did not amplify any contaminating mRNA. The primer sequence of *UROS* and *MIR4484* DNA primers used is as follows: Uros genomic FP: CCATCGGAAATTGCTTAGGA, Uros genomic RP: CAGGCCCCTTGACTCAGTAG, MIR4484 genomic FP: GAGGCTTGAGACTGGTGAGG, MIR4484 genomic RP: GCCGAGGTGAGTTTCATGTT. The *C*_t_ values of *UROS* and *MIR4484* were normalized with the *C*_t_ value of GAPDH (DNA gene content) to obtain Δ*C*_t_. The final copy number was deduced by ΔΔ*C*_t_ method. All qPCRs were run on ABI PRISM 7900HT Sequence Detection System (Life Technologies, ThermoFisher), and thermal cycling conditions were 95 °C for 15 min followed by 40 cycles of 95 °C for 25 s, 60 °C for 30 s and 72 °C for 30 s, followed by dissociation curve analysis.

### 2.4 RNA extraction, cDNA conversion and quantitative RT‐PCR analysis

Total cellular RNA that was inclusive of the miRNA fraction was extracted using Trizol reagent (Sigma) as per the manufacturer's instructions. RNA thus isolated was subjected to quality check and analysed for its purity and integrity by Nano‐drop ND‐1000 Spectrophotometer (Nano‐Drop Technologies, Wilmington, DE, USA) and gel electrophoresis. For cDNA synthesis, 2 μg of total RNA was taken and subjected to conversion using ‘High capacity cDNA reverse transcription kit’ (Applied Biosystems, ThermoFisher) according to the manufacturer's protocol. cDNA synthesized was eventually diluted with nuclease‐free water in the ratio of 1 : 10 such that the final concentration of the cDNA was 10 ng·μL^−1^. Expression of *Uros* and other genes was assayed by SYBR green‐based real‐time quantitative PCR carried out in the ABI PRISM 7900HT Sequence Detection System (Applied Biosystems) under default conditions: 95 °C for 15 min, 40 cycles of 95 °C for 15 s, 60 °C for 20 s and 72 °C for 25 s. Analysis of gene expression was performed using the ΔΔ*C*_T_ method (Reddy *et al*., 2008) wherein *GAPDH*,* 18S rRNA* and *RPL35A* genes were used as internal controls for data normalization. The primer sequence of *UROS*,* GAPDH*,* 18S rRNA* and *RPL35A* mRNA primers used is as follows: Uros FP: GGAGAAACCTGTGGAAATGC, Uros RP: GCAATCCCTTTGTCCTTGAG, GAPDH FP: TTGTCAAGCTCATTTCCTGG, GAPDH RP: TGATGGTACATGACAAGGTGC, 18S rRNA FP: GTAACCCGTTGAACCCCATT, 18S rRNA RP: CCATCCAATCGGTAGTAGCG, RPL35A FP: ACGCCCGAGATGAAACAG, RPL35A RP: GGGTACAGCATCACTCGG. The primer oligonucleotides were ordered from Sigma‐Aldrich (St. Louis, MO, USA).

### 2.5 Real‐time qPCR for quantification of miRNAs

We employed an LNA‐based system for the sensitive and accurate detection and estimation of miRNAs by quantitative real‐time PCR using SYBR Green. The method involves the synthesis of universal cDNA followed by real‐time quantitative PCR amplification with LNA‐enhanced primers (Exiqon Inc., Vedbaek, Denmark). Real‐time PCR was performed using the ABI PRISM 7900 HT Sequence Detection System (Life Technologies). The thermal profile of the real‐time qPCR is as follows: 95 °C for 10 min and then 45 cycles of 95 °C for 10 s and 60 °C for 1 min with a ramp rate of 1.6 °C·s^−1^. U6, U44 and U48 small RNAs were used in different combinations as endogenous controls. ΔΔ*C*_T_ method was used for the calculation of expression ratios.

### 2.6 Pre‐miR mimics transfection of glioma cells

In order to overexpress the downregulated miRNAs, the precursors or mimics of corresponding miRNA were transfected into the glioma cell lines. The pre‐miR mimics and Cy‐3‐labelled pre‐miR control/unlabelled pre‐miR‐negative controls were procured from Ambion. We employed a reverse transfection method for transfecting pre‐miRs and their respective negative controls, wherein the transfection mix is added to an empty dish to begin with and then the dish is overlaid with the requisite volume of cell suspension (Nawaz *et al*., 2016). siPORT transfection reagent (Ambion) was first diluted in a requisite volume of OptiMEM medium (reduced serum medium), and this mix was incubated at room temperature for 10 min. Meanwhile, the pre‐miRs and their respective pre‐miR‐negative controls (10 μm stock) were also diluted at a particular concentration in OptiMEM. The diluted siPORT and pre‐miR were mixed together after 10 min of incubation and again incubated at room temperature for another 10 min. The mix was finally added to an empty dish wherein the cell suspension was subsequently overlaid at a desired cell density. The final concentration of pre‐miRs and their respective pre‐miR‐negative controls in the culture medium was 50 nm. The dish was incubated in a CO_2_ incubator. RNA was isolated after 24 h of pre‐miR transfection, and the overexpression of miRNA was confirmed by assaying for the level of mature miRNA by LNA‐based quantitative real‐time PCR as explained previously (Nawaz *et al*., 2016).

### 2.7 Colony suppression assay

The colony‐forming ability of cells upon miRNA overexpression was gauged by transfection of negative control pre‐miR or pre‐miR‐4484 in glioma cells. Cells were harvested after 24 h of transfection and counted in each group. Around 3000 or 6000 cells were plated in duplicate from each group in a 12‐well cluster plate to assay for colony formation. The culture media was changed every three days, and the growth of the cells was monitored till they attained a significant colony size. After two to three weeks of plating, the colonies were fixed in chilled methanol for 30 min and stained with 0.05% crystal violet solution for another 30 min and photographed.

### 2.8 Wound healing scratch assay

To evaluate the migratory potential of glioma cells upon miR‐4484 transfection, cells were transfected with negative control pre‐miR or pre‐miR‐4484. After 24 h of transfection, cells were counted and seeded in a 12‐well cluster plate in duplicate at a high density to form a monolayer. After 12–24 h, the monolayer was mechanically disrupted with a sterile toothpick to produce a clean uniform scratch mimicking a wound. The wells were photographed every 12 h, and the gap distances were recorded at each time point to assess the closure of the wound.

### 2.9 Transcriptome analysis by RNA sequencing

The quality and quantity of RNA samples were assessed using Agilent's Bioanalyser. For each sample, we used 1 μg of total cellular RNA for library preparation. Samples were run in duplicate to obtain the data. TrueSeq RNA sample preparation kit (Cat # RS‐122‐2001) was used as per the manufacturer's guidelines to generate library for sequencing. The above‐generated library was re‐quantified using Agilent's Bioanalyser and through real‐time qPCR methodology. From each sample library, 120 μL (10 pm) was taken for subsequent workflow. The strands were denatured and subjected to cluster generation on the flow cell in the c‐Bot system using TruSeq PE Cluster kit (Cat # PE‐401‐3001). The flow cell was finally subjected to two rounds of sequencing (Read1 and Read2), and the results were obtained as intensity files. The sequencing was carried out on Illumina HiScanSQ, using Truseq SBS V3 technology for 50‐bp paired‐end reads RNA sequencing (Cat # FC‐401‐3002). tophat (version 2.0.10) was used to align the raw reads on the human reference genome (hg19). After the alignment step, the aligned files were subjected to cufflink (version 2.2.0) to generate a transcriptome assembly. Cuffmerge utility was used to merge all the assemblies to generate the final transcriptome assembly. This transcriptome assembly was subsequently subjected to Cuffquant utility to quantify gene expression. Normalization of quantified gene expression data was carried out using Cuffnorm utility. The differentially expressed genes were finally identified using Cuffdiff utility. Genes that had an absolute fold change greater than 1.5 with FDR‐adjusted *P*‐value < 0.05 were considered significant.

### 2.10 Other data sets

To compare and corroborate the expression of miR‐4484 and other genes evaluated in the study, the fold change of the mentioned genes was further derived from various publicly available data portals, including TCGA data set (Agilent and Affymetrix platforms), REMBRANDT and GSE22866 data set.

### 2.11 Bioinformatics analysis

miRWalk (http://zmf.umm.uni-heidelberg.de/apps/zmf/mirwalk2/), an online miRNA target prediction tool that provides the data compiled from 12 prediction algorithms, was used to predict the gene targets for miR‐4484.

### 2.12 Gene ontology analysis

DAVID (Database for Annotation, Visualization and Integrated Discovery) (https://david.ncifcrf.gov/) Bioinformatics Resources tool was used for gene ontology mining and pathway analysis.

### 2.13 Statistical analysis

The statistical significance between two groups was calculated by unpaired Student's t‐test using the graphpad prism software (GraphPad PRISM, La Jolla, CA, USA). In the experimental cases dealing with more than two groups, the statistical significance was calculated by one‐way ANOVA (analysis of variance) with Tukey's *post hoc* test using the graphpad prism software. The statistical significance obtained is symbolically indicated as asterisks (*). *P*‐value of < 0.05 was considered to be statistically significant, (ns) not significant; (*) *P* < 0.05; (**) *P* < 0.01 and (***) *P* < 0.001.

## 3 Results

### 3.1 Somatic copy number alterations distort the genetic landscape of miRNAs and influence their expression

**Figure 1:**
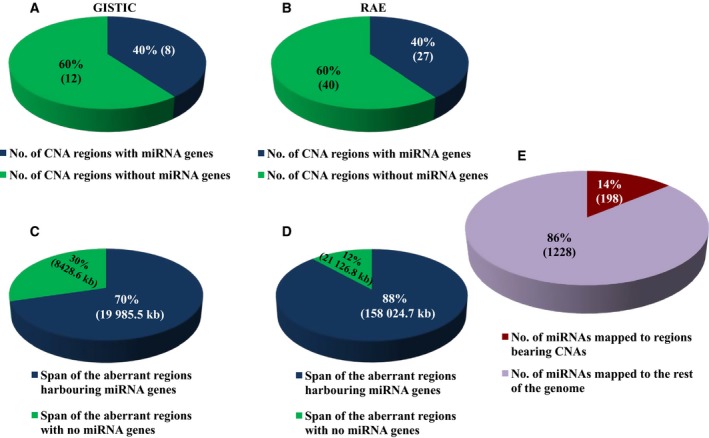
Overview of the miRNAs in relation to copy number aberrations. (A,B) Pie chart depicting the percentage number of aberrant events in GBM bearing the miRNA sites in both GISTIC‐ and RAE‐derived identifiers. (C,D) Pie chart depicting the percentage span in terms of length of aberrant regions bearing the miRNA sites in both GISTIC‐ and RAE‐derived identifiers. (E) Percentage number of miRNAs that are localized to the regions bearing copy number alterations as against the total number of miRNAs, depicted as a pie chart.

In order to investigate the impact of genomic deletions and amplifications on the miRNome, we used TCGA copy number data and analysed the regions exhibiting aberrant copy number behaviour, as identified through GISTIC (Genomic Identification of Significant Targets in Cancer) and RAE (Relative Absolute Error) algorithms. While GISTIC identified 20 aberrant events, RAE algorithm identified 67 such events (Table S1). Using bioinformatics approach, we mapped the miRNAs to these aberrant genetic loci. Cumulatively, we were able to map miRNAs to 8 of 20 aberrant loci identified by GISTIC and 27 of 67 aberrant loci identified by RAE (Table S1). Thus, 40% of such copy number altered loci harboured miRNA genes in both GISTIC‐ and RAE‐derived aberrant regions, indicating the frequent localization of miRNAs in such fragile genomic loci (Fig. 1A,B). Further, we investigated the aberrant regions in terms of their size or genomic span. This analysis revealed that about 40% of the aberrant events that bore the miRNA genes actually covered 70–88% span of the aberrant genome (Fig. 1C,D). Since the minor percentage of aberrant events bearing miRNA genes covered a majority of the aberrant genome, it surmised that most of the miRNA genes are enriched in the larger aberrant genomic segments which undergo deletions or amplifications and thus are more susceptible to genomic alterations rendering them fragile in nature. Additionally, we also found that about 14% of the total number of miRNAs (198 miRNAs of 1426 mature miRNAs) localize to such fragile aberrant genomic regions. This indicates that a significant proportion of the aberrant genome carries miRNA genes, thereby underscoring the importance of copy number events as the important source for the derailed miRNome in GBM (Fig. 1E).

**Figure 2:**
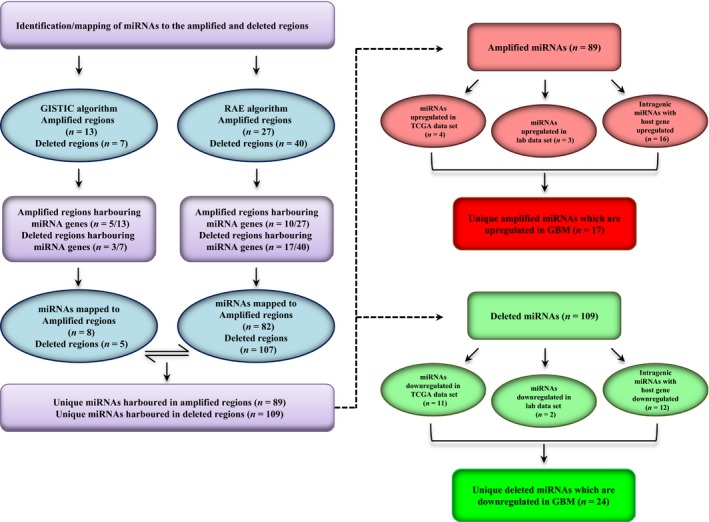
*In silico* identification of miRNAs located in the somatic copy number‐altered regions in GBM. Flowchart describing the analysis pipeline for the shortlisting of copy number‐altered miRNAs.

Further, to identify precisely the miRNAs that are truly misregulated owing to the alterations in the copy number status of the malignant cell, we sorted the aberrant regions in both GISTIC and RAE into amplified and deleted regions. We identified 13 amplified and seven deleted genomic loci in GISTIC‐derived regions and 27 amplified and 40 deleted genomic loci from RAE identifiers, and all of them did not harbour miRNA genes as mentioned previously (Fig. 2). The mapped miRNAs were subsequently classified as amplified and deleted region‐derived miRNAs depending upon their location. Cumulatively from GISTIC‐ and RAE‐derived aberrant identifiers, we were able to deduce 89 unique miRNA located in the amplified regions and 109 unique miRNAs located in the deleted regions (Fig. 2; Tables S2 and S3). Further, we shortlisted the miRNAs based on their expression and/or their host gene expression in the context of their genetic alteration. In other words, miRNAs mapped to deleted regions were expected to have lesser expression and *vice versa*. We shortlisted 17 miRNAs that were located in the amplified regions and were either upregulated themselves or at the level of their host genes (Fig. 2; Table 1). Similarly, 24 miRNAs were either downregulated or their host gene had lesser expression and were harboured in the regions with genomic deletions (Fig. 2; Table 2). miR‐4484 was taken ahead for further validation and characterization as it was mapped to a genomic region, which was among the most deleted regions on chromosome 10, with 130 and 91 samples having a segment mean of < −0.3 and < −0.5, respectively (Table S3).

**Table 1:** List of miRNAs located in the regions with genomic amplification, their regulation in GBM and the relevant information of their host gene

S.No	miRNA	Region hg19	TCGA		Lab Data^a^		Host gene		
			Diff. LFC^b^	Adj. *P* (Benjamini–Hochberg)	Diff. LFC^b^	*P*‐value	Gene	Diff. LFC^b^	Adj. *P* (Benjamini–Hochberg)
1	hsa‐miR‐339	chr7:229794‐12384994	1.10	0.000004	NA	NA	C7orf50	0.20	0.294596
2	hsa‐miR‐93	chr7:97568276‐100324915	1.49	0.000002	2.03	0.000000	MCM7	0.96	0.000004
3	hsa‐miR‐106b	chr7:97568276‐100324915	1.87	0.000002	2.36	0.000000	MCM7	0.96	0.000004
4	hsa‐miR‐25	chr7:97568276‐100324915	1.90	0.000002	2.23	0.000000	MCM7	0.96	0.000004
5	hsa‐miR‐3125	chr2:12061625‐20964134	NA	NA	NA	NA	TRIB2	1.20	0.000176
6	hsa‐miR‐4648	chr7:229794‐12384994	NA	NA	NA	NA	LFNG	1.17	0.000328
7	hsa‐miR‐3609	chr7:97568276‐100324915	NA	NA	NA	NA	TRRAP	0.68	0.000217
8	hsa‐miR‐3973	chr11:32434754‐37349348	NA	NA	NA	NA	LDLRAD3	1.52	0.000002
9	hsa‐miR‐26a‐2	chr12:58075399‐58311523	NA	NA	NA	NA	CTDSP2	0.91	0.000046
10	hsa‐miR‐4745	chr19:113418‐2800481	NA	NA	NA	NA	PTBP1	1.50	0.000002
11	hsa‐miR‐199a‐1	chr19:10070230‐11643726	NA	NA	NA	NA	DNM2	0.92	0.000003
12	hsa‐miR‐4748	chr19:10070230‐11643726	NA	NA	NA	NA	DNM2	0.92	0.000003
13	hsa‐miR‐638	chr19:10070230‐11643726	0.22	0.597781	−1.69	0.002120	DNM2	0.92	0.000003
14	hsa‐miR‐3189	chr19:18148615‐19986025	NA	NA	NA	NA	GDF15	1.79	0.000020
15	hsa‐miR‐640	chr19:18148615‐19986025	0.15	0.000243	0.30	0.686010	GATAD2A	0.98	0.000005
16	hsa‐miR‐643	chr19:52589104‐54795358	0.03	0.002225	NA	NA	ZNF766	0.65	0.009546
17	hsa‐miR‐4754	chr19:56623806‐59092515	NA	NA	NA	NA	RPS5	0.92	0.000116

^a^ Lab Data: Genome‐wide expression profiling identifies deregulated miRNAs in malignant astrocytoma (Rao *et al*., 2010).

^b^ Diff LFC: differential log_2_ fold change (GBM minus control brain).

**Table 2:** List of miRNAs located in the regions with genomic deletions and their regulation in GBM and the relevant information of their host gene

S.No	miRNA	Region hg19	TCGA		Lab Data^a^		Host gene		
			Diff. LFC^b^	Adj. *P* (Benjamini–Hochberg)	Diff. LFC^b^	*P*‐value	Gene	Diff. LFC^b^	Adj. *P* (Benjamini–Hochberg)
1	hsa‐miR‐134	chr14:101464256‐102072149	−0.59	0.000088	0.42	0.287537	NA	NA	NA
2	hsa‐miR‐154	chr14:101464256‐102072149	−0.71	0.000002	NA	NA	NA	NA	NA
3	hsa‐miR‐323	chr14:101464256‐102072149	−1.41	0.000002	NA	NA	NA	NA	NA
4	hsa‐miR‐377	chr14:101464256‐102072149	−1.51	0.000003	0.08	0.739883	NA	NA	NA
5	hsa‐miR‐379	chr14:101464256‐102072149	−1.88	0.000002	−0.33	0.230623	NA	NA	NA
6	hsa‐miR‐381	chr14:101464256‐102072149	−1.11	0.000002	0.44	0.001586	MIR381HG	NA	NA
7	hsa‐miR‐410	chr14:101464256‐102072149	−2.20	0.000002	−2.00	0.000006	NA	NA	NA
8	hsa‐miR‐411	chr14:101464256‐102072149	−1.03	0.000002	−0.03	0.889330	NA	NA	NA
9	hsa‐miR‐487a	chr14:101464256‐102072149	−0.64	0.000020	−0.25	0.209734	NA	NA	NA
10	hsa‐miR‐487b	chr14:101464256‐102072149	−0.95	0.000008	−0.40	0.236795	MIR381HG	NA	NA
11	hsa‐miR‐758	chr14:101464256‐102072149	−0.87	0.000002	NA	NA	NA	NA	NA
12	hsa‐miR‐495	chr14:101464256‐102072149	−0.53	0.000002	−0.65	0.005893	NA	NA	NA
13	hsa‐miR‐2681	chr13:79820643‐115105760	NA	NA	NA	NA	FGF14	−1.34	0.010560
14	hsa‐miR‐4705	chr13:79820643‐115105760	NA	NA	NA	NA	FGF14	−1.34	0.010560
15	hsa‐miR‐1233‐1	chr15:33089320‐41016345	NA	NA	NA	NA	GOLGA8A	−1.15	0.002720
16	hsa‐miR‐1233‐2	chr15:33089320‐41016345	NA	NA	NA	NA	GOLGA8A ;GOLGA8B	−1.15	0.002720
17	hsa‐miR‐1286	chr22:16124218‐20311704	NA	NA	NA	NA	RTN4R	−3.55	0.000002
18	hsa‐miR‐3944	chr10:127622813‐135506681	NA	NA	NA	NA	ECHS1	−0.93	0.000002
19	hsa‐miR‐4296	chr10:127622813‐135506681	NA	NA	NA	NA	CTBP2	−0.61	0.000027
20	hsa‐miR‐4484	chr10:127622813‐135506681	NA	NA	NA	NA	UROS	−1.50	0.000004
21	hsa‐miR‐4501	chr13:79820643‐115105760	NA	NA	NA	NA	HS6ST3	−3.65	0.000002
22	hsa‐miR‐4535	chr22:42957116‐51218950	NA	NA	NA	NA	FAM19A5	−1.27	0.000002
23	hsa‐miR‐4762	chr22:42957116‐51218950	NA	NA	NA	NA	ATXN10	−0.70	0.000010
24	hsa‐miR‐648	chr22:16124218‐20311704	0.19	0.031338	NA	NA	MICAL3	−0.94	0.000021

^a^ Lab Data: Genome‐wide expression profiling identifies deregulated miRNAs in malignant astrocytoma (Rao *et al*., 2010).

^b^ Diff LFC: differential log_2_ fold change (GBM minus control brain).

### 3.2 miR‐4484 and its host gene UROS exhibit a similar genomic deletion pattern in GBM

**Figure 3:**
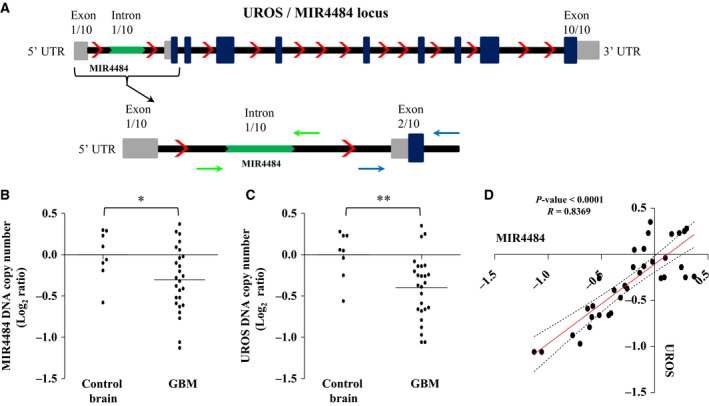
miR‐4484 undergoes copy number loss in GBM. (A) Schematic depicting the genomic location of MIR4484 gene in the intronic region of *UROS* gene. Grey bars represent UTRs, while blue broad and black thinner bars represent exons and introns, respectively. Reds arrows, demarcated all along the stretch, indicate the directionality of the gene. Green arrowed bar represents the mature miRNA region embedded in the first intron. Paired forward and reverse arrows of same colour indicate the primer pairs encompassing MIR4484 (precursor sequence) and the second exon of *UROS*. (B,C) Scatter plot indicating the DNA copy number (log_2_ ratio) of MIR4484 and *UROS*, primers specific to the MIR4484 and *UROS* locus were used for the quantitative DNA copy number PCR. (D) The genomic amplification (copy number) values obtained from the DNA qPCR were used to assess the correlation in the copy number of *UROS* and MIR4484. A high positive correlation, with a correlation coefficient *R* = 0.8369 and *P *<* *0.0001, was obtained between the two in terms of their abundance. *P < 0.05 and **P < 0.01.

miR‐4484 is an intragenic miRNA that resides in the first intron of the *uroporphyrinogen III synthase* (*UROS*) gene located on the positive strand of chromosome 10 (Fig. 3A). In order to establish that miR‐4484 is subjected to genomic deletion in GBM, we carried out a copy number DNA qPCR. We demonstrated that indeed MIR4484 locus undergoes copy number loss by assaying the genomic levels of the region encompassing miRNA precursor sequence (Fig. 3B). Moreover, we also assayed the copy number levels of its host gene UROS, and observed that it was also significantly deleted in GBM as compared to control brain (Fig. 3C). As the DNA qPCR for MIR4484 and UROS was carried out in a similar set of samples, we were able to correlate the deletion pattern between them. We observed that the two events were closely linked to each other as there was a high significant positive correlation between the two (Fig. 3D). Thus, our results indicate that the codeletion of Uros and miR‐4484 is a closely linked event in GBM.

### 3.3 Genomic deletion of miR‐4484 leads to its downregulation in GBM

**Figure 4:**
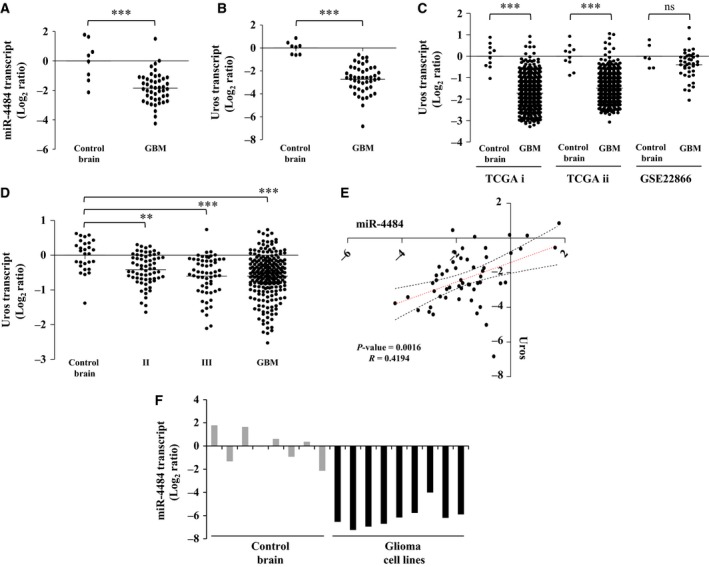
miR‐4484 and its host gene Uros are downregulated in GBM. (A,B) Log_2_‐transformed miR‐4484 and *Uros* expression values obtained from qRT‐PCR analysis indicating their downregulation in GBM, respectively. Each dot represents the data derived from one sample. For each sample, fold change in expression is calculated over its average expression in control brain tissue. (C) Log_2_‐transformed *Uros* expression ratios obtained from TCGA i (Affymetrix), TCGA ii (Agilent) and GSE22866 data sets indicating significant downregulation of *Uros* in GBM. (D) Log_2_‐transformed *Uros* expression ratios across different grades of glioma, obtained from the REMBRANDT data set indicating its significant downregulation from earlier grades. (E) The transcript expression values obtained from the qRT‐PCR experiment were used to assess the correlation in the expression of *Uros* and miR‐4484 in our sample cohort. A high positive correlation, with a correlation coefficient *R* = 0.4194 and *P* = 0.0016, was obtained. (F) Log_2_‐transformed miR‐4484 expression ratios across different glioma cell lines, SVG, T98G, LN18, LN229, U87, U138, U251, U343 and U373 (respectively from left to right, black solid columns) as compared to control brain tissue (grey solid columns). *P‐*values were calculated by Student's *t*‐test and one‐way ANOVA; the symbols indicated are explained as follows: *P < 0.05; **P < 0.01 and ***P < 0.001.

Further, to investigate the effect of genomic deletion on the expression of miR‐4484, we assayed its transcript levels. We measured the transcript levels of mature miR‐4484 in our sample cohort and observed that indeed miR‐4484 was significantly downregulated in GBM as compared to control brain samples (Fig. 4A). Additionally, we also observed that *Uros* transcript levels were also downregulated in GBM in the same sample cohort (Fig. 4B). This finding was further corroborated by the fact that *Uros* was also downregulated in other publicly available data sets TCGA, GSE22866 and the REMBRANDT data set (Fig. 4C,D). Next, to confirm whether there existed any relationship between the expression of miR‐4484 and its host gene *Uros*, we performed expression correlation analysis. We found that there was a significant positive correlation in the transcript levels of miR‐4484 and *Uros* in our GBM sample cohort (Fig. 4E). We also measured the levels of miR‐4484 in glioma cell lines and found it to be significantly downregulated in all the cell lines, compared to control brain (Fig. 4F). Thus, our results indicate that the deletion of UROS locus results in the downregulation of both the host gene and miR‐4484.

In order to verify whether the deletion at the UROS locus affected only the host gene UROS and MIR4484, we assayed the levels of the two genes *BRCA2 and CDKN1A interacting protein* (BCCIP) and *matrix metallopeptidase 21* (MMP21), lying in the immediate vicinity of UROS on either side (Fig. S1A). BCCIP is located upstream to UROS (at a distance of ~ 0.3 kb) but on the positive strand, while as MMP21 resides downstream to UROS (at a distance of ~ 13 kb) on the same negative strand. The expression in the TCGA data sets and GSE22866 indicated that BCCIP did not show any significant downregulation; on the contrary, BCCIP was upregulated in one of the TCGA platforms and GSE22866 (Fig. S1B). MMP21 did show downregulation in the TCGA data set (Affymetrix platform) but was not regulated in the GSE22866 data set, while as its expression data was not available from the Agilent platform of TCGA (Fig. S1C). Moreover, we also assayed the expression of BCCIP and MMP21 in our sample cohort to find out their expression pattern. We observed that both BBCIP and MMP21 were not regulated in GBM compared to control brain (Fig. S1D,E). Thus, we conclude that the focal deletion event at UROS locus affects the expression of *Uros* and miR‐4484 exclusively, underscoring the importance of the event.

### 3.4 miR‐4484 overexpression inhibits the colony formation and the migration capacity of glioma cells

**Figure 5:**
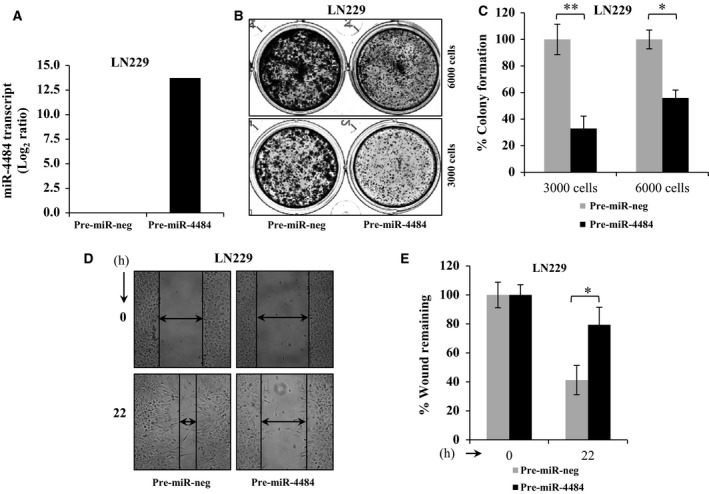
miR‐4484 overexpression exerts tumour‐suppressive effects in GBM. (A) Log_2_‐transformed miR‐4484 expression values obtained from qRT‐PCR analysis upon its overexpression in LN229 cell line. (B,C) LN229 cells were transfected with pre‐miR‐negative control (pre‐miR‐neg) or pre‐miR‐4484 at a final concentration of 50 nm; 3000 and 6000 cells were plated in duplicate in a 12‐well cluster plate. After three weeks of plating, the colonies were stained with crystal violet, photographed (left) and counted (right). % Colony density ± SD is plotted. (D,E) LN229 cells transfected with pre‐miR‐neg or pre‐miR‐4484 were subjected to scratch assay (left); potential of migrating capacity of cells was quantitated by the percentage of wound remaining after indicated time point. *P‐*values were calculated by Student's *t*‐test, and the symbols indicated are explained as follows: *P < 0.05.

miR‐4484 being deleted and downregulated in GBM, we overexpressed it by pre‐miR‐4484 transfection and evaluated its effect on various parameters that are relevant to the tumorigenic phenotype. The pre‐miR‐4484 transfection indeed resulted in high levels of mature miR‐4484 in LN229 glioma cells (Fig. 5A). We observed that the reintroduction of miR‐4484 significantly reduced the colony‐forming potential of glioma cells (Fig. 5B,C). In addition, we also observed that the overexpression of miR‐4484 significantly hindered the migration of cells as the cells failed to fill in the gap in a scratch assay (Fig. 5D,E). The data collectively indicate that the exogenous overexpression of miR‐4484 imparts tumour‐suppressive effects in glioma cells, thus underscoring this deletion as an essential event in gliomagenesis.

### 3.5 miR‐4484‐regulated target genes suggest its role in gliomagenesis

**Figure 6:**
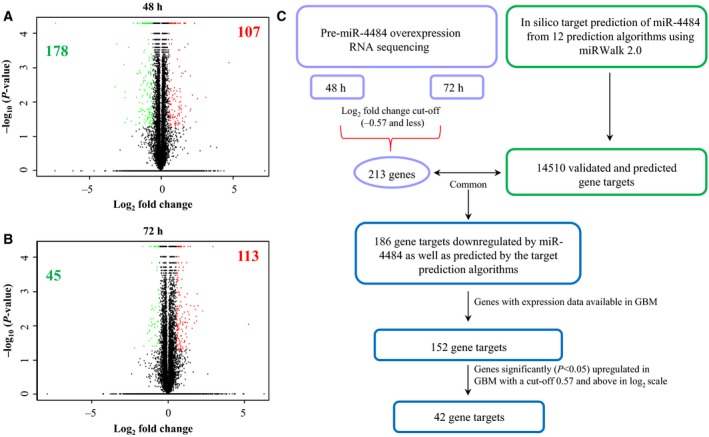
Transcriptome profile upon miR‐4484 overexpression. (A,B) Volcano plot representation of the RNA sequencing data, indicating the genes significantly and differentially expressed between pre‐miR‐neg and pre‐miR‐4484 condition in LN229 cell line at two 48‐ and 72‐h time points, respectively. Each red dot indicates a gene significantly upregulated in pre‐miR‐4484 overexpression condition, and each green dot indicates a gene significantly downregulated in pre‐miR‐4484 overexpression condition. The figures (bolded) inside each plot denote the number of genes upregulated and downregulated significantly. Green indicates the downregulated genes, and red indicates the upregulated genes. (C) Schematic representation of miRNA target prediction. All the genes downregulated (−0.57 and less in the log_2_ scale) upon miR‐4484 overexpression from both 48‐ and 72‐h time point were sought for the analysis. miR‐4484 targets were derived from 12 target prediction algorithms (miRWalk, MicroT4, miRanda, miRBridge, miRDB, miRMap, miRNAMap, PICTAR2, PITA, RNA22, RNAhybrid and Targetscan) assembled in miRWalk 2.0 online tool. The experimental targets were compared with the *in silico* targets to find out the common ones, which were finally compared for their expression in GBM.

Further, to elucidate the putative targets of miR‐4484 and to establish its role in GBM, we overexpressed miR‐4484 exogenously by pre‐miR transfection and analysed the changes in the transcriptome by RNA sequencing at two different time points (48 and 72 h) in LN229 glioma cell line. In fact, we observed a vast number of genes being regulated upon miR‐4484 overexpression as depicted in the volcano plots (Fig. 6A,B). The genes regulated by miR‐4484 were shortlisted further based on their fold change and *P*‐value significance. We found 107 genes to be upregulated and 178 genes downregulated upon pre‐miR‐4484 transfection at the 48‐h time point (Table S4). Also, 113 genes were found to be upregulated and 45 genes downregulated at the 72‐h time point (Table S5). To select and validate the direct potential targets of miR‐4484, we focussed on the list of downregulated genes obtained through RNA sequencing and employed an integrated bioinformatics approach as explained in the schematic (Fig. 6C). The differential transcriptome from two different time points (48 and 72 h) was assessed separately and we shortlisted 213 downregulated genes (cut‐off −0.57 and less in the log_2_ scale), owing to the fact that the direct putative targets of miR‐4484 should get downregulated upon miR‐4484 overexpression (Fig. 6C). In parallel, we also fetched the *in silico* gene targets of miR‐4484 using miRWalk 2.0, an online web tool that extracts and generates data cumulatively from 12 prediction algorithms (Fig. 6C). A list of 186 genes, predicted to have miR‐4484 binding site *in silico* and downregulated upon miR‐4484 overexpression, were narrowed down for further study (Fig. 6C). The putative targets of miR‐4484 were eventually shortlisted based on their expression in GBM compared to the control brain. As miR‐4484 is downregulated in GBM, we hypothesized that the gene targets of miR‐4484 which were downregulated upon pre‐miR‐4484 transfection should be upregulated in GBM. A list of 42 such genes was observed to be significantly upregulated in GBM (Fig. 6C; Table S6). This list of genes represents the potential targets of miR‐4484 in GBM.

**Figure 7:**
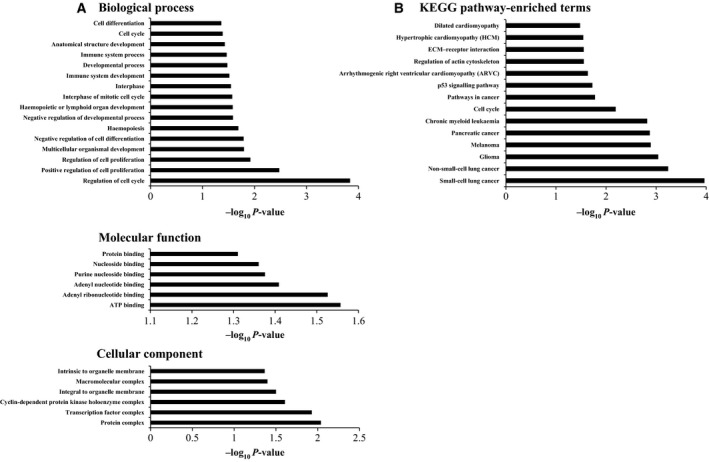
Gene ontology and pathways analysis of the miR‐4484 transcriptome. (A) Analysis of annotation category ‘Gene ontology’ indicating the different terms being enriched for the significantly downregulated genes carrying the miR‐4484 target sites. (B) Analysis of annotation category ‘Pathways’ indicating the different terms being enriched in the KEGG annotation source, proposing to the role of miR‐4484 in gliomagenesis.

In order to ascertain the function of miR‐4484 and attribute the phenotype we observed upon its overexpression, we carried out an equitable functional enrichment analysis of its potential targets (genes that were downregulated upon miR‐4484 overexpression and carried the targets sites for miR‐4484). The gene ontology and pathways annotation categories in DAVID exhibited a significant enrichment of the terms such as regulation of cell cycle, cell proliferation, cell differentiation and mitosis in the ‘gene ontology’ annotation category (Fig. 7A). In the ‘pathways’ annotation category, we observed significant enrichment of terms in the Kyoto Encyclopedia of Genes and Genomes annotation source, which included some important terms such as p53 signalling pathway, ECM–receptor interaction, pathways in cancer, and different types of cancers including glioma (Fig. 7B). All these specifically enriched terms cumulatively propose the role of miR‐4484 in producing the observed phenotype.

**Figure 8:**
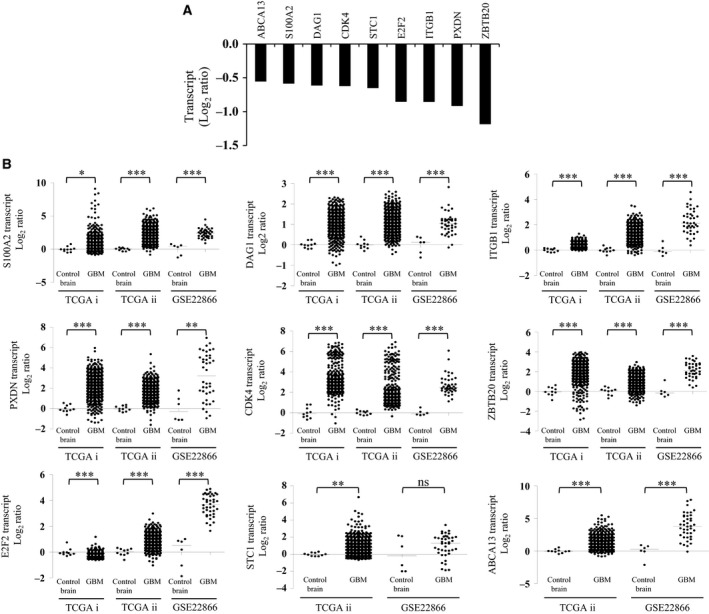
Validation of the putative miR‐326 targets. (A) The column plot depicts the transcript levels of the putative targets of miR‐4484 upon pre‐miR‐4484 transfection, validated through qRT‐PCR analysis. (B) Transcript analysis of the putative targets of miR‐4484 in GBM. Data are derived from TCGA and GSE22866 data sets; TCGA i and TCGA ii refer to Affymetrix and Agilent platforms, respectively. *P‐*values were calculated by Student's *t*‐test, and the symbols indicated are explained as follows: ^ns^not significant; *P < 0.05; **P < 0.01 and ***P < 0.001.

Finally, from the potential gene target list, we validated nine genes that include *ABCA13*,* S100A2*,* DAG1*,* CDK4*,* STC1*,* E2F2*,* ITGB1*,* PXDN* and *ZBTB20*, which were downregulated to varying extents upon pre‐miR‐4484 transfection (Fig. 8A). To further corroborate our assumption of these genes as the targets of miR‐4484, we analysed the transcript levels of these genes across different publicly available GBM data sets (TCGA and GSE22866) and observed that most of them were significantly upregulated in GBM, laying further thrust for these genes to be the potential targets of miR‐4484 (Fig. 8B).

## 4 Discussion

The misregulation of miRNAs has added one more realm of intricacy and complexity to the comprehension of tumour biology. miRNAs play a crucial role in the maintenance of homeostasis by refining the expression of protein‐coding genes. Glioblastoma or the grade IV astrocytoma is no exception and is significantly influenced by the misregulation of miRNAs (Karsy *et al*., 2012; Rao *et al*., 2010). There are many studies that have reported the aberrant expression of miRNAs in GBM (Ciafre *et al*., 2005; Rao *et al*., 2010). miRNAs although do not encode any proteins but they can certainly function as potential oncogenes and tumour suppressors, thereby invoking a paradigm change in the normal functioning of a cell. Alterations in the levels of miRNAs have been linked to almost all facets of cancer biology, including cell proliferation, migration, angiogenesis, and chemoresistance (Esquela‐Kerscher and Slack, 2006; Garzon *et al*., 2006).

The misregulation of miRNAs could result as a consequence of a wide array of aberrant cellular phenomena such as chromosomal translocations, transcriptional activation due to a novel transcription factor getting hyperactivated, amplification or deletions in a locus and epigenetic modifications. Copy number aberrations are one of the potent distorters of the genome as they inevitably alter the dose of a particular gene product available to a cell. Somatic copy number alterations are well known for their activation of oncogenes and deactivation of tumour suppressor genes in various malignancies including GBM. Beroukhim *et al*. (2007) identified 16 broad and 16 focal events in GBM, which harboured some of the crucial oncogenes and tumour suppressor genes such as TP53, RB1, CDKN2A/B, PTEN, EGFR, PDGFRA, MET, CDK4, CDK6, MDM2, MDM4 and MYC and some novel to GBM such as MYCN, PIK3CA, CCND2, KRAS and CHD5 (Beroukhim *et al*., 2007). TCGA group also unveiled some rare focal events like amplifications of FGFR2 and IRS2, and deletion of PTPRD which though being infrequent were quite informative of their role in tumorigenesis (Bartel, 2004, 2008). Moreover, the same study brought into picture some alterations that were not previously known, like the homozygous deletions of NF1 and PARK2 and amplification of AKT3 (Bartel, 2004, 2008). A similar picture is quite elaborately known for other cancers as well. In B‐cell chronic lymphocytic leukaemia, miR‐15 and miR16a were observed to be located in the region 13q14, which was deleted in about 65% of the patients, and the allelic loss correlated with the downregulation of both the miRNAs (Calin *et al*., 2002). It was further demonstrated that miRNA genes often venture into the fragile sites of the genome, like the regions involving amplifications, break points, loss of heterozygosity and other cancer‐associated genomic regions (Calin *et al*., 2004). This study investigated 186 miRNA genes and observed that 98 of the miRNA genes were harboured in such fragile loci (Calin *et al*., 2004). Another pan‐cancer study indicated that the genomic loci harbouring miRNAs genes exhibited high proportion of somatic copy number alterations accounting for about 37.1% in ovarian cancer, 72.8% in breast cancer and 85.9% in melanomas (Zhang *et al*., 2006). miRNA genes were also demonstrated to undergo substantial SCNAs in lung cancer, with miR‐30d, miR‐21, miR‐17 and miR‐155 being the most amplified ones (Czubak *et al*., 2015).

In this study, we observed that indeed the miRNA genes localize to the fragile regions of the GBM genome, which makes them susceptible to changes in their abundance on account of deletions and amplifications. Thus, it lays a thrust on the coincidence of miRNA genes being localized to such fragile genomic loci. In fact, it indicates their importance and qualifies them to be one of the drivers of tumorigenesis. We observed that around 40% of the copy number aberrant events harbour miRNA genes and most of the miRNA genes were located in the larger deleted or amplified fragments. We speculate that larger genomic events represent the loci which undergo copy number alterations more frequently and might be more fragile. Our investigation revealed that a significant number of miRNAs were influenced by such genomic aberrations.

We also demonstrated a novel intragenic miRNA, miR‐4484 being deleted in GBM. Moreover, we validated that miR‐4484 is downregulated in GBM as compared to control brain samples and its downregulation is attributed to the genomic copy number loss of its genetic locus. Moreover, a recent study also indicated the downregulation of miR‐4484 in diffuse large B‐cell lymphoma (Tamaddon *et al*., 2016). We also observed that the deletion of miR‐4484 was accompanied by the deletion of its host gene *Uros*, and the loss of both the gene and the miRNA was a tightly linked codeletion event in GBM. In fact, we observed a unique behaviour in some samples in terms of the expression of host gene and the miRNA, which both showed no copy number loss in a few samples in a unified way, indicating a strong association between the two and the deletion always encompassing either both or none. In other words, we did not come across a sample where there was discordance in the copy number of miRNA and its host gene.

Although miR‐4484 is located in the first intronic region of the Uros gene, they do not share the same strand. Moreover, *Uros* has not been implicated in any malignancy, so it suggests that the two are regulated independent of each other. Overexpression of miR‐4484 inhibited the colony formation and also delayed the migration of glioma cells. We also identified S100A2, DAG1, ITGB1, PXDN, CDK4, ZBTB20, E2F2, STC1 and ABCA13 as the potential targets of miR‐4484. Most of these genes are upregulated in GBM and were also predicted by different target prediction algorithms, thereby putting thrust on their authenticity as genuine targets of miR‐4484. We also validated these gene targets of miR‐4484 at the level of their transcripts upon miR‐4484 overexpression. It is noteworthy that most of these genes play a significant role in gliomagenesis in particular as well as in other cancers in general. S100A2 has been reported to be hypomethylated in GBM (Etcheverry *et al*., 2010; Martinez and Schackert, 2007) and functions as an inducer of metastasis *in vivo* in non‐small‐cell lung cancer (Bulk *et al*., 2009). Overexpression of S100A2 also induced epithelial‐to‐mesenchymal transformation followed by enhanced invasion and increased AKT phosphorylation in A549 lung cancer cells (Naz *et al*., 2014). Similarly, ZBTB20 has been found to be an important poor prognostic factor in hepatocellular carcinoma owing to its elevated levels in HCC and importantly, metastatic recurrence rates were found to be higher in HCC patients with high ZBTB20 levels (Wang *et al*., 2011). ABCA13 was found to be upregulated in colorectal cancer (Hlavata *et al*., 2012), and its high expression was related to less overall survival in metastatic ovarian serous carcinoma (Nymoen *et al*., 2015). Another target of miR‐4484, STC1, also exhibited relatively higher expression in high‐grade gliomas as compared to low‐grade samples (Su *et al*., 2015). Furthermore, samples with high expression of STC1 had worse overall survival as against samples with low STC1 expression (Su *et al*., 2015). E2F2, an E2F family transcription factor, has been related to various phenotypes such as cell proliferation, cell cycle, apoptosis and DNA damage response. E2F2 has been reported to be a poor prognostic marker in NSCLC. Expression of E2F2 was found to be markedly high in NSCLC samples as compared to normal specimens and was closely associated with clinical stage and tumour size (Chen *et al*., 2015). E2F2 was also observed to be upregulated in astrocytomas, and more importantly, it exhibited a distinctive expression in CD133‐positive cells which are considered to be enriched in glioma stem‐like cells (Okamoto *et al*., 2007). CDK4 is a well‐known oncogene in GBM, and in one of the landmark studies, CDK4 was found to be the second most amplified gene in GBM after EGFR, and this focal amplification eventually leads to its high expression in GBM (Bartel, 2004, 2008). PXDN, yet another putative gene target of miR‐4484, is an adhesion biomolecule which functions as an important regulator of cellular plasticity and extracellular matrix remodelling. PXDN levels were found to be elevated in primary glial and secondary metastatic brain tumours as well as in its immediate microvasculature (Liu *et al*., 2010). Similarly, ITGB1, a cell adhesive molecule, which functions to mediate association between cells and the ECM, has also been found to be misregulated in various cancers and found to impact malignant phenotypes such as invasion, proliferation and angiogenesis (He *et al*., 2016; Zhang and Zou, 2015). A study also suggested the role of ITGB1 in the secretion and activation of MMP14 (Mori *et al*., 2013). In brief, most of these genes have a well‐characterized role in GBM or other human malignancies and moreover, being upregulated in GBM, it can be conceived that miR‐4484 brings about its tumour‐suppressive effects by targeting these genes. Gene ontology and pathway analysis also indicated the role of these genes in various cancer‐related processes and terms as was evidenced in the phenotype we observed. Taken together, all these data underscore the importance of the focal deletion of miR‐4484 in GBM.

## Author contributions

KS, VS, AA and ASH coordinated the study; ZN, SR and KS conceived the study. ZN designed, performed and analysed all the experiments. VP and SA did the computational analysis of the RNA sequencing data and other statistical analysis including data acquisition. ZN and KS drafted and revised the article critically for important intellectual content. All authors reviewed the results and approved the final version of the manuscript to be submitted.

## Supporting information


**Fig. S1.** Genomic deletion at MIR4484 locus is a specific deletion event in GBM that exclusively affects Uros and miR‐4484.

**Table S1.** List of copy number altered (amplified and deleted regions) identified by GISTIC and RAE algorithms and the regions harbouring miRNA genes among them.

**Table S2.** List of miRNAs mapped to the amplified genetic loci in the glioma genome.

**Table S3.** List of miRNAs mapped to the deleted genetic loci in the glioma genome.

**Table S4.** Genes regulated by miR‐4484 overexpression at 48 h interval (Cut‐off ± 0.57 in Log_2_ scale).

**Table S5.** Genes regulated by miR‐4484 overexpression at 72 h interval (Cut‐off ± 0.57 in Log_2_ scale).

**Table S6.** List of genes downregulated upon miR‐4484 overexpression, upregulated in GBM and bearing binding sites for miR‐4484.
