# Supplementary material for: Impact of somatic copy number alterations on the glioblastoma miRNome: miR‐4484 is a genomically deleted tumour suppressor
Source: Mol Oncol. 2017 May 24;11(8):927–44. doi: 10.1002/1878-0261.12060 (PMC5537698; doi:10.1002/1878-0261.12060)
Supplement: Supplementary file 1 — Fig. S1. Genomic deletion at MIR4484 locus is a specific deletion event in GBM that exclusively affects Uros and miR‐4484. [file MOL2-11-927-s001.pptx]

## Slide 1
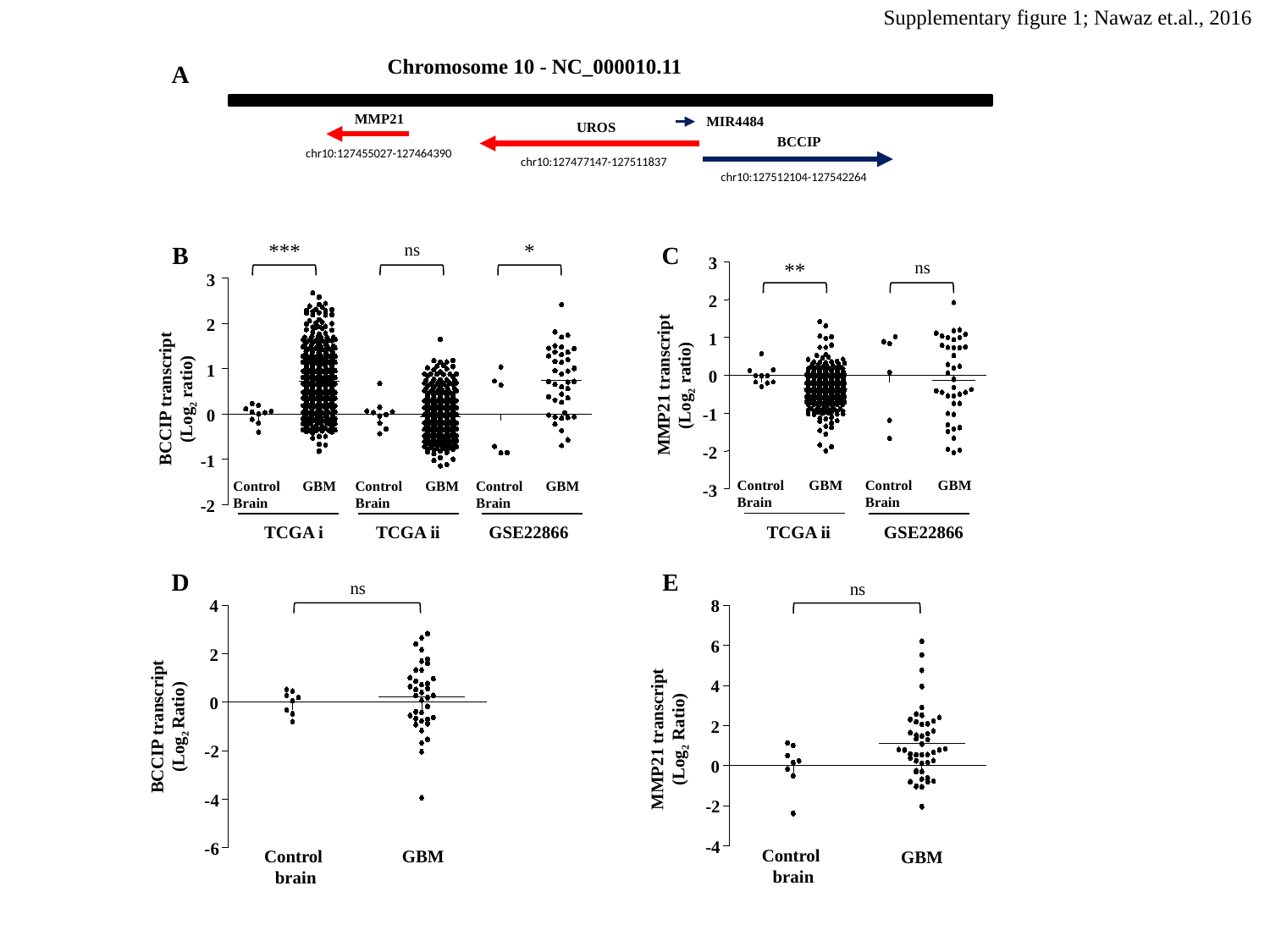

Supplementary figure 1; Nawaz et.al., 2016
Chromosome 10 - NC_000010.11
MMP21
MIR4484
UROS
BCCIP
chr10:127455027-127464390
chr10:127477147-127511837
chr10:127512104-127542264
A
***
*
ns
3
2
1
0
-1
-2
BCCIP transcript
(Log2 ratio)
 TCGA i TCGA ii GSE22866
Control
Brain
GBM
Control
Brain
GBM
Control
Brain
GBM
3
2
1
0
-1
-2
-3
MMP21 transcript
(Log2 ratio)
ns
**
 TCGA ii GSE22866
Control
Brain
GBM
Control
Brain
GBM
B
C
D
E
ns
4
2
0
BCCIP transcript
(Log2 Ratio)
-2
-4
-6
Control
 brain
GBM
ns
8
6
4
MMP21 transcript
(Log2 Ratio)
2
0
-2
-4
Control
brain
GBM
