# Supplementary material for: Impact of somatic copy number alterations on the glioblastoma miRNome: miR‐4484 is a genomically deleted tumour suppressor
Source: Mol Oncol. 2017 May 24;11(8):927–44. doi: 10.1002/1878-0261.12060 (PMC5537698; doi:10.1002/1878-0261.12060)
Supplement: Supplementary file 8 [file MOL2-11-927-s008.docx]

**Supplementary Information**

**Supplementary Figure 1. Genomic deletion at MIR4484 locus is a specific deletion event in GBM that exclusively affects Uros and miR-4484.**

**A.** Log_2_-transformed expression ratios obtained from TCGA i (affymetrix), TCGA ii (agilent) and GSE22867 data sets indicating no significant downregulation of bccip in GBM.

**B.** Log_2_-transformed expression ratios obtained from TCGA ii (agilent) and GSE22867 data sets indicating downregulation of mmp-21 being downregulated in TCGA and not regulated in GSE22867.

**C, D.** Log_2_-transformed bccip and mmp-21 expression ratios obtained from real-time qRT-PCR analysis. bccip and mmp-21 are not regulated in GBM. Each dot represents the data derived from one sample. For each sample, fold change in expression is calculated over its average expression in normal brain tissue.

*p* values were calculated by student’s t-test and the symbols indicated are explained as follows: (ns) not significant; (*) p ≤ 0.05; (**) p ≤ 0.01 and (***) p≤0.001
